# Reconsidering “Water‐Based” Classification in Poly‐L‐Lactic Acid Vascular Safety

**DOI:** 10.1111/jocd.71286

**Published:** 2026-11-09

**Authors:** Chuan‐Yuan Lin, Jui‐Yu Lin

**Affiliations:** ^1^ Nanzi Annyeong Clinic Kaohsiung Taiwan; ^2^ Taipei Royalty Aesthetic Clinic Taipei Taiwan; ^3^ Chengdu Royalty Aesthetic Clinic Chengdu Sichuan China; ^4^ Hangzhou Royalty Aesthetic Clinic Hangzhou Zhejiang China

**Keywords:** carboxymethylcellulose, poly‐L‐lactic acid, rheology, Sculptra, vascular occlusion, vascular safety

To the Editor,

We read with interest the article by Lam et al. describing clinical experience with Sculptra (poly‐L‐lactic acid; PLLA‐SCA) and expert recommendations for minimizing complications [1]. The authors provide useful guidance across patient assessment, treatment planning, product preparation, and procedural safety. Within this broader consensus, we wish to comment on one specific statement regarding vascular safety. Based on their clinical experience, the panel suggests that PLLA‐SCA may have a lower risk of vascular occlusion, including retinal artery occlusion, because it is a water‐based suspension rather than a gel‐based biostimulator [1]. Importantly, reconstituted PLLA‐SCA is an aqueous particulate suspension containing PLLA microparticles together with carboxymethylcellulose (CMC) and mannitol. Each vial contains 150 mg of PLLA, 90 mg of CMC, and 127.5 mg of mannitol [2]. CMC is a water‐soluble polymer whose aqueous solutions exhibit concentration‐dependent rheological behavior [3]. Thus, an aqueous carrier designation describes the solvent system but does not by itself define the rheological state of the reconstituted formulation.

Reconstitution volume necessarily alters the nominal CMC concentration and may therefore alter the rheological behavior of the preparation. Published protocols have evolved from approximately 3 mL of sterile water for facial treatment to 8 mL of sterile water plus 1 mL of lidocaine [4]. Based on 90 mg of CMC per vial, these preparations correspond to nominal concentrations of 3.0% and 1.0% w/v, respectively. The 9‐mL preparation also reflects the protocol used by Lam et al. [1]. Intermediate calculated concentrations are summarized in Table 1. In aqueous CMC solutions, Benchabane and Bekkour demonstrated a steep concentration‐dependent increase in zero‐shear viscosity and identified transitions near 1% and 2.5% by weight, with increasingly viscoelastic behavior above the latter concentration [3]. Nevertheless, these material‐specific thresholds cannot be assumed for PLLA‐SCA, particularly because their concentrations were expressed by weight rather than as the nominal weight/volume percentages used here.

**TABLE 1:** Added liquid volume, nominal CMC concentration, and supporting evidence for PLLA‐SCA.

Added liquid volume per vial	Nominal CMC concentration^a^	Preparation context and supporting evidence
3 mL SWFI	3.0% w/v	Historical low‐volume facial preparation described by Vasconcelos‐Berg et al. [4]
5 mL SWFI	1.8% w/v	Earlier 5‐mL reconstitution protocol cited by Baumann et al. [5]
7 mL SWFI	1.29% w/v	Laboratory preparation studied by Li et al. before any lidocaine addition [2]
8 mL SWFI	1.13% w/v	Baumann et al. reported mean viscosities of 10.5–11.0 mPa s for the filtered aqueous fraction after removal of undissolved particles [5]
9 mL total	1.0% w/v	8 mL SWFI plus 1 mL lidocaine, as used by Lam et al. The 8‐mL viscosity measurements are not directly applicable to this 9‐mL preparation [1, 5]

*Note:* Aqueous CMC reference data: For the CMC grade studied by Benchabane and Bekkour (nominal molecular weight 700 kDa; degree of substitution 0.65–0.85; measurement temperature 20°C), Cross‐model zero‐shear viscosities were 0.53 Pa·s at 1%, 1.97 Pa·s at 1.5%, 6.36 Pa·s at 2%, 21 Pa·s at 2.5%, and 48 Pa·s at 3% (all wt%) [3]. These weight‐based values and the reported transition concentrations are not directly transferable to PLLA‐SCA. Reference numbers correspond to the manuscript.

Abbreviations: CMC, carboxymethylcellulose; PLLA‐SCA, poly‐L‐lactic acid (Sculptra); SWFI, sterile water for injection; w/v, weight/volume; wt%, weight percentage.

^a^ Nominal values are calculated from 90 mg CMC per vial and the stated added‐liquid volume, not a measured final suspension volume [2]. Any subsequently added liquid should be included in the denominator.

In a product‐specific study, Baumann et al. reported mean viscosities of 10.5–11.0 mPa·s after reconstitution with 8 mL of sterile water. Their methods specify removal of undissolved particles by 0.2‐μm filtration before viscosity testing [5]. These measurements therefore characterize the filtered aqueous fraction rather than the complete particulate suspension. Notably, these values are substantially below the 0.53 Pa·s (530 mPa·s) zero‐shear viscosity reported for aqueous CMC at 1% by weight [3, 5]. However, sample preparation and rheometric conditions differed, and equivalence of the CMC grades was not established. Baumann et al. reported a shear test rate range of 10–10 000 s^−1^, whereas Benchabane and Bekkour derived zero‐shear viscosities from Cross‐model fits [3, 5]. Therefore, the discrepancy cannot be assigned to a single cause, nor can the aqueous CMC series predict PLLA‐SCA viscosity. Experimental vascular models further suggest that filler rheology can influence intravascular fragmentation, dispersal, and occlusive behavior. In an artificial arterial embolism model, Soares and McCarthy found differences in HA–gel fragmentation, dispersal, and branch occlusion among products with differing rheological characteristics, with tan (δ) showing the strongest relationship with fragment size [6]. Beyond the continuous phase, PLLA‐SCA also contains a dispersed solid phase. Its particles have been characterized as heterogeneous microflakes approximately 2–150 μm in major‐axis length [7]. General embolization literature indicates that particle size, shape, aggregation, compressibility, and elasticity can influence embolic behavior [8]. A simulated microvascular study of calcium hydroxylapatite‐CMC (CaHA‐CMC), another particle‐containing aesthetic injectable, demonstrated distal occlusion and retrograde propagation [9]. These experimental findings cannot be directly extrapolated to PLLA‐SCA, but they support the broader principle that an aqueous carrier designation alone is insufficient to characterize intravascular behavior.

The panel reported no blindness cases related to PLLA‐SCA in Malaysia [1]. However, serious ocular vascular complications following PLLA‐SCA injection have been reported elsewhere. These include retinal artery occlusion after temporal injection and severe visual loss with orbital infarction after periorbital injection [10, 11]. Accordingly, although the authors appropriately recognize that vascular risk is multifactorial [1], assigning a lower vascular occlusion risk to PLLA‐SCA primarily on the basis of its “water‐based” classification has not been established by direct comparative evidence. An aqueous carrier designation alone does not establish comparative vascular safety. Product‐specific rheological and vascular studies may help test the proposed explanation, but any clinical safety advantage requires comparative evidence involving the relevant alternative formulations.

## Funding

The authors have nothing to report.

## Ethics Statement

The authors have nothing to report.

## Conflicts of Interest

The authors declare no conflicts of interest.

## Data Availability

The authors have nothing to report.
